# Supplementary material for: Multiplexed transcriptome discovery of RNA-binding protein binding sites by antibody-barcode eCLIP
Source: Nat Methods. 2022 Dec 22;20(1):65–9. doi: 10.1038/s41592-022-01708-8 (PMC9834051; doi:10.1038/s41592-022-01708-8)
Supplement: Supplementary file 1 — Supplementary Figs. 1 and 2. [file 41592_2022_1708_MOESM1_ESM.pdf]

nature methods

Brief Communication https://doi.org/10.1038/s41592-022-01708-8

# Multiplexed transcriptome discovery of RNA-binding protein binding sites by antibody-barcode eCLIP

In the format provided by the authors and unedited

Supplementary Figure 1.

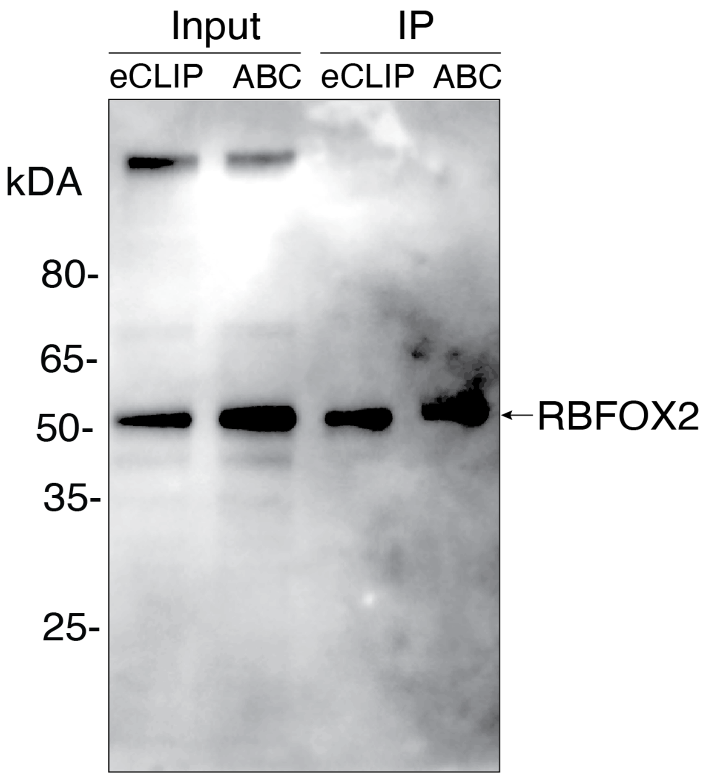

Supplementary Figure 2.

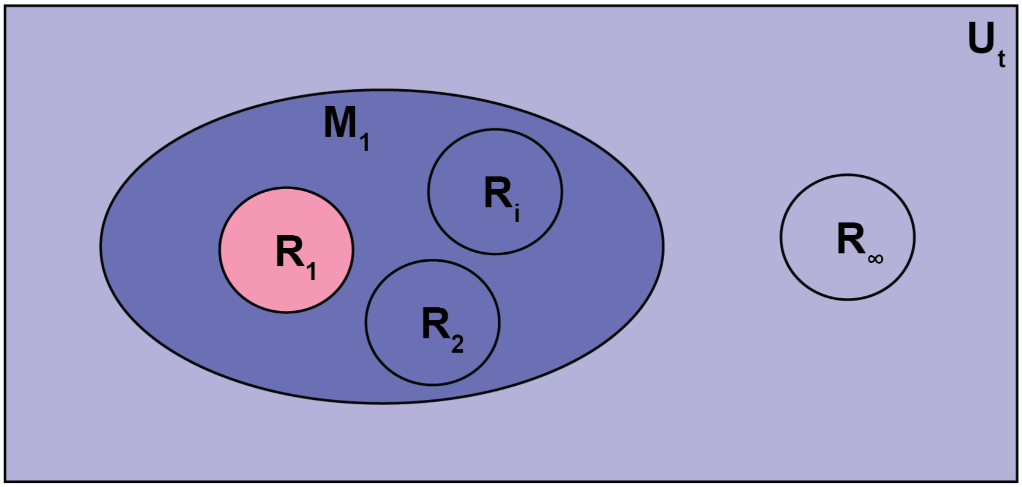


We assume that for a given RBP, $R_1$, that exists within the universe (U) of cell type ($U_t$) that a multiplex set ($M_1$) = [$R_1$, $R_2$, ..., $R_i$] containing a number (i) of distinct RBPs will serve as a complement ($R_1$') as "i" approaches all possible RBPs (∞). Hence, we define using multiplexed RBPs as an input control as "complement control" (CC). Ideally, $R_1$ should be within $M_1$, however, this may not be necessary.

Supplementary Fig. 1: Immunoprecipitation of RBFOX2 from HEK293XT using normal (eCLIP) and barcoded (ABC) antibodies. This experiment was performed as a single replicate.

Supplementary Fig. 2: Description and visualization of the definition of complement control.

Supplementary Table 1: Top 100 genes enriched by SLBP ABC and eCLIP (Fig.1g & Supplementary Fig.7).

Supplementary Table 2: Number of reads for each RBP in each 10-plex ABC experiment in K562 cells.

Supplementary Table 3: List of antibodies and barcodes used.

**Supplementary Note 1. Calculation of the conserved motif**
Conserved motifs within 3'UTR or intronic regions contained a score phyloP[33] score > 3.

**Supplementary Note 2. Guidance for pooling and sequencing depth**
We have observed varying amounts of reads assigned to each barcode, despite pooling an equal number of beads and antibodies. This is likely due to each RBP having varying levels of expression, expression of a given RBP's target RNAs, and the effectiveness of the IP antibody for that RBP. Future users should be mindful that antibodies need to be CLIP grade and, at this time, we suggest empirically tested and adjusted if equal coverage is required. Based on eCLIP/ENCODE guidelines we found that sequencing each sample following the formula (# of barcodes X 25 Million reads) provided sufficient depth of each RBP to recover their known biological binding preferences. However, for many RBPs this is insufficient depth to reach complete saturation of binding sites. Each RBP will have to be empirically tested to determine the correct sequencing depth to reach complete saturation.
