# Supplementary material for: Multiplexed transcriptome discovery of RNA-binding protein binding sites by antibody-barcode eCLIP
Source: Nat Methods. 2022 Dec 22;20(1):65–9. doi: 10.1038/s41592-022-01708-8 (PMC9834051; doi:10.1038/s41592-022-01708-8)
Supplement: Supplementary file 2 — Reporting Summary [file 41592_2022_1708_MOESM2_ESM.pdf]

nature portfolio

Corresponding author(s): Daniel A. Lorenz, Hsuan-Lin Her, Kylie A. Shen, Katie Rothamel, Kasey R. Hutt, Allan C. Nojadera, Stephanie C. Bruns, Sergei A. Manakov, Brian A. Yee, Karen B. Chapman, Gene W. Yeo

Last updated by author(s): Oct 17, 2022

Reporting Summary

Nature Portfolio wishes to improve the reproducibility of the work that we publish. This form provides structure for consistency and transparency in reporting. For further information on Nature Portfolio policies, see our Editorial Policies and the Editorial Policy Checklist.

Statistics

For all statistical analyses, confirm that the following items are present in the figure legend, table legend, main text, or Methods section.

n/a	Confirmed	
☐	☒	The exact sample size (*n*) for each experimental group/condition, given as a discrete number and unit of measurement
☐	☒	A statement on whether measurements were taken from distinct samples or whether the same sample was measured repeatedly
☐	☒	The statistical test(s) used AND whether they are one- or two-sided<br>*Only common tests should be described solely by name; describe more complex techniques in the Methods section.*
☒	☐	A description of all covariates tested
☐	☒	A description of any assumptions or corrections, such as tests of normality and adjustment for multiple comparisons
☐	☒	A full description of the statistical parameters including central tendency (e.g. means) or other basic estimates (e.g. regression coefficient) AND variation (e.g. standard deviation) or associated estimates of uncertainty (e.g. confidence intervals)
☐	☒	For null hypothesis testing, the test statistic (e.g. *F*, *t*, *r*) with confidence intervals, effect sizes, degrees of freedom and *P* value noted<br>*Give P values as exact values whenever suitable.*
☐	☒	For Bayesian analysis, information on the choice of priors and Markov chain Monte Carlo settings
☐	☒	For hierarchical and complex designs, identification of the appropriate level for tests and full reporting of outcomes
☐	☒	Estimates of effect sizes (e.g. Cohen's *d*, Pearson's *r*), indicating how they were calculated

Our web collection on statistics for biologists contains articles on many of the points above.

Software and code

Policy information about availability of computer code

Data collection

Data was processed similarly to the standard eCLIP pipeline, except for a few adjustments to ABC's multiplex design and library structure. For ABC data, unique molecular identifiers (UMI) were extracted using umitools, and adaptors were removed using cutadapt. Fastqs files were demultiplexed based on the 5' nucleotide barcode sequence using fastx toolkit (http://hannonlab.cshl.edu/fastx_toolkit/). ABC libraries were sequenced on the reverse strand. Therefore, reads were reverse complemented before alignment to repetitive regions, removal of multi-mapped reads, and alignment to the genomic sequences using STAR. The pipeline is available at https://github.com/algaebrown/oligoCLIP.git.
UMI-tools version: 1.0.0
cutadapt 2.8
fastx_toolkit: 0.0.14
fastq_tools: 0.8
fastQC: 0.11.8
STAR: 2.7.6a
samtools: 1.6
clipper: 2.1.2

Data analysis

All code for figures can be found here: https://github.com/algaebrown/oligoCLIP.git

For manuscripts utilizing custom algorithms or software that are central to the research but not yet described in published literature, software must be made available to editors and reviewers. We strongly encourage code deposition in a community repository (e.g. GitHub). See the Nature Portfolio guidelines for submitting code & software for further information.

March 2021

Data

Policy information about availability of data

All manuscripts must include a data availability statement. This statement should provide the following information, where applicable:

- Accession codes, unique identifiers, or web links for publicly available datasets
- A description of any restrictions on data availability
- For clinical datasets or third party data, please ensure that the statement adheres to our policy

All code for analysis is accessible here https://github.com/algaebrown/oligoCLIP.git. eCLIP data used to compare against can be found here: https://www.encodeproject.org/eclip/
Encode HEPG2 RNA-seq data: https://www.encodeproject.org/experiments/ENCSR245ATJ/
Encode K562 RNA-seq data: https://www.encodeproject.org/experiments/ENCSR615EEK/

ABC data is available at GEO accession: GSE205536

Human research participants

Policy information about studies involving human research participants and Sex and Gender in Research.

Reporting on sex and gender	N/A
Population characteristics	N/A
Recruitment	N/A
Ethics oversight	N/A

Note that full information on the approval of the study protocol must also be provided in the manuscript.

Field-specific reporting

Please select the one below that is the best fit for your research. If you are not sure, read the appropriate sections before making your selection.

☒ Life sciences ☐ Behavioural & social sciences ☐ Ecological, evolutionary & environmental sciences

For a reference copy of the document with all sections, see nature.com/documents/nr-reporting-summary-flat.pdf

Life sciences study design

All studies must disclose on these points even when the disclosure is negative.

Sample size	No sample size calculations were performed. Encoded data was uploaded as duplicates and used as is. ABC data was performed in at least duplicates to match ENCODE standards.
Data exclusions	There are no data exclusions.
Replication	All replicates are included. eCLIP experiments were performed with two experimental replicates. ABC experiments performed with either two (singleplex) or three (multiplex) replicates.
Randomization	Allocation of experimental groups was not random, covariates, such as RNA expression levels, were controlled by the following: eCLIP experiment was normalized against a size matched input, singleplex ABC experiments underwent normalization against total RNA-seq/(rRNA-depleted RNA-seq when total RNA-seq is not available) whereas multiplexCLIP data normalize against both total RNA-seq and internal normalization using a chi squared test/fisher exact test between the other 9 RBPs in the multiplex.
Blinding	Because we were comparing the data of a new method to a previously establish method (eCLIP), blinding is not relevant to our experiment.

Reporting for specific materials, systems and methods

We require information from authors about some types of materials, experimental systems and methods used in many studies. Here, indicate whether each material, system or method listed is relevant to your study. If you are not sure if a list item applies to your research, read the appropriate section before selecting a response.

Materials & experimental systems

n/a	Involved in the study
☐	☒ Antibodies
☐	☒ Eukaryotic cell lines
☒	☐ Palaeontology and archaeology
☒	☐ Animals and other organisms
☒	☐ Clinical data
☒	☐ Dual use research of concern

Methods

n/a	Involved in the study
☒	☐ ChIP-seq
☒	☐ Flow cytometry
☒	☐ MRI-based neuroimaging

Antibodies

Antibodies used

RBFOX2 Bethyl A300-864A
PUM2 Bethyl A300-202A
DDX3 Bethyl A300-474A
FAM120A Bethyl A300-899A
ACH11A Bethyl A300-524A
LIN28B Bethyl A300-588A
SF3B4 Bethyl A300-950A
EIF3G Bethyl A300-755A
PRPF8 Bethyl A300-921A
IGF2BP2 MBL RN008P
SLBP Bethyl A300-968A

Validation

Each antibody is searchable at this link: https://www.encodeproject.org/search/?type=AntibodyLot&status=released and was validated using the guidelines at this link: https://www.encodeproject.org/documents/fb70e2e7-8a2d-425b-b2a0-9c39fa296816/@@download/attachment/ENCODE_Approved_Nov_2016_RBP_Antibody_Characterization_Guidelines.pdf in addition to manufactures validation.

Eukaryotic cell lines

Policy information about cell lines and Sex and Gender in Research

Cell line source(s)

K562 (Homo sapiens, adult 53 years, female) - ATCC
HEK293XT (Homo sapiens, embryonic, female) - Takara Bio

Authentication

Outside of the authentic commercial source, no authentication of cell lines were used.

Mycoplasma contamination

Cells were not tested for mycoplasma contamination.

Commonly misidentified lines
(See ICLAC register)

No commonly misidentified lines were used in this study.
